# Foundations for a realist ontology of mental disease

**DOI:** 10.1186/2041-1480-1-10

**Published:** 2010-12-09

**Authors:** Werner Ceusters, Barry Smith

**Affiliations:** 1 Ontology Research Group, Center of Excellence in Bioinformatics and Life Sciences, 701 Ellicott street, Buffalo, NY 14203, USA; 2 Department of Psychiatry, University at Buffalo, NY, USA; 3 Department of Philosophy, University at Buffalo, NY, USA

## Abstract

While classifications of mental disorders have existed for over one hundred years, it still remains unspecified what terms such as 'mental disorder', 'disease' and 'illness' might actually denote. While ontologies have been called in aid to address this shortfall since the GALEN project of the early 1990s, most attempts thus far have sought to provide a formal description of the structure of some pre-existing terminology or classification, rather than of the corresponding structures and processes on the side of the patient.

We here present a view of mental disease that is based on ontological realism and which follows the principles embodied in Basic Formal Ontology (BFO) and in the application of BFO in the Ontology of General Medical Science (OGMS). We analyzed statements about what counts as a mental disease provided (1) in the research agenda for the DSM-V, and (2) in Pies' model. The results were used to assess whether the representational units of BFO and OGMS were adequate as foundations for a formal representation of the entities in reality that these statements attempt to describe. We then analyzed the representational units specific to mental disease and provided corresponding definitions.

Our key contributions lie in the identification of confusions and conflations in the existing terminology of mental disease and in providing what we believe is a framework for the sort of clear and unambiguous reference to entities on the side of the patient that is needed in order to avoid these confusions in the future.

## Introduction

### The Classification of Diseases

There is a long history of attempts to categorize what are commonly referred to as 'diseases', 'disorders' or 'illnesses'. These include the development of a series of classification systems, of which the International Classification of Diseases (ICD) developed by the World Health Organization (WHO) is the oldest and most widely used [1]. The underlying presupposition of such categorial systems is that, for purposes of morbidity and mortality reporting, *patients with the same type of disease *should be classified or categorized by means of the *same disease code*. This would enable data formulated in terms of such classifications to be used for purposes such as comparing the incidence and prevalence of diseases across institutions, jurisdictions, healthcare systems, and so forth. It would also allow patients to be stratified into homogenous groups in relation to which the outcomes of given therapeutic interventions could be reliably assessed.

It is Kraepelin who is responsible for introducing into psychiatric diagnosis a *categorical approach to classification *along these lines [2], an approach which views mental disorders as organized into discrete clinical categories. Kraepelin was working at a time when few technical means were available to assist the diagnostic process, so that clinicians had to rely on overt symptoms and signs exhibited by the patient that were believed to be manifestations of an underlying disorder. Some of these, whether taken in isolation or in combination with others, were referred to as '*marker*' symptoms and signs, and were believed to be pathognomonic for the disorder in question, which means that they were considered to be so characteristic for the disorder that their presence alone would render a diagnosis definitive. Such pathognomonic markers were then contrasted with those signs and symptoms that provided weaker diagnostic signals and were thus seen as requiring in addition consideration of the patient's individual constitution.

Nowadays the most prominent categorical classification system for mental disorders - where for the moment we are leaving aside the question of the distinction in meaning, if any, between 'disorder' and 'disease' - is of course the *Diagnostic and Statistical Manual of Mental Disorders *(DSM) [3] published by the American Psychiatric Association (APA). The DSM provides diagnostic criteria to guide the clinician in making a diagnosis, together with additional information on how to differentiate the instances of any given disorder from those instances of other disorders that have similar presenting characteristics. This criterion-based approach is also applied in the chapter on mental disorders of ICD-10 (in contrast to what is the case in all other chapters of ICD, which merely list the various subcategories for each disease). In addition, WHO provided ICD-10 with two distinct diagnostic guidelines for mental disorders: one for clinical use [4], and one for research purposes [5]. These guidelines are intended to reduce the variability in coding caused by two sorts of disagreement which can arise when diagnoses are being made:

(1) differences in opinion amongst clinicians about what type of mental disorder a patient with a certain configuration of symptoms and test results is suffering from - in this case the disagreement is about the diagnosis itself, independent of the diagnostic options offered by the ICD or DSM;

(2) differences in opinion about which ICD or DSM classification code should be used even in those cases where there is agreement about a diagnosis.

### What is a mental disorder?

While ICD and DSM have each provided multiple elaborate systems for the classification of mental disease, the more fundamental question, concerning what a mental disease/disorder/illness actually *is*, has still not been answered in a satisfactory way [6]. It is this more fundamental question that we shall address in the present paper.

Szasz has argued provocatively that '*mental illness is a myth whose function it is to disguise and thus render more palatable the bitter pill of moral conflicts in human relations*' [7]. Contrary to some popular misconceptions of his views, Szasz, or so we assume, did not mean that none of the disorders subsumed by the term 'mental illness' exist. Rather he held that the group of persons '*known to manifest various peculiarities or disorders of thinking and behavior*' and about which it is therefore said that they have a mental illness, can be divided into two subgroups:

(1) those for which there is an underlying *brain disorder *perhaps not yet discoverable by what the state of the art is able to offer, examples being patients with Alzheimer disease or with depression associated with Parkinson's disease;

(2) those without brain disorder who exhibit in their behavior a '*deviance ... from certain psychosocial, ethical, or legal norms*' as judged by themselves, by clinicians, or by others, for example people preferring solitude or for whom family relationships are not important while living in a society that values company and family very highly.

Those in group (1), according to Szasz, would be better described as having a brain disorder, while those in group (2), while they might indeed have '*problems of living*', and thus be suffering, are not suffering because of some disorder of a special, mental kind. Szasz hereby rejects as fallacious the view which regards social intercourse '*as something inherently harmonious, its disturbance being due solely to the presence of "mental illness" in many people*'.

Others, such as Jablensky, raise the question whether psychiatry really needs an overarching and universal definition of 'mental disorder' at all [8], and it is indeed a fact that neither WHO nor the APA provide definitions for this term. The 'Lexicon of psychiatric and mental health terms', which was prepared by WHO to accompany the mental disorder chapters of ICD-9 and ICD-10, says only that 'mental disorder' is '*An imprecise term designating any disorder of the mind, acquired or congenital*' [9]. The more specific term 'organic mental disorder' it defines as '*a range of mental disorders grouped together on the basis of their having in common a demonstrable etiology in cerebral disease, brain injury, or other insult, leading to cerebral dysfunction*'. The lexicon provides no clue as to whether WHO rules out the existence of mental disorders which are not due to some disorder of the brain, and it is in any case stated in the ICD-10 chapter on mental disorders that the term 'disorder' is used rather loosely [4]. It is thus no surprise that the associated research agenda for the new edition (DSM-V) of the *Diagnostic and Statistical Manual*, scheduled for release in May 2013, calls for the generation of acceptable definitions for '*mental disorder*', '*disease*' and '*illness*' [10]. In what follows, we present the foundations of an ontology of mental disease that is intended to contribute to the formulation of such definitions.

## Background

Ontologies are created to serve multiple goals, including: supporting effective retrieval of data, reducing the detrimental effects of silo formation in biomedical databases caused by differences in the underlying information models and terminologies used, and allowing different sorts of formal reasoning. Sadly, while developing ontologies has become over the past decade a popular activity, the lack of shared and well-tested principles for ontology development has resulted in a multitude of ontology-like artifacts of different sorts, whose effect has been to exacerbate, rather than solve, the problem of data silos. An exception is the Open Biomedical Ontologies (OBO) Foundry [11] initiative, which accepts under its label only those ontologies that adhere to the principles of ontological realism [12]. Where the prevailing, i.e. computer science, view of ontology is focused on the logical consistency and inferential implications of ontologies as sets of assertions, the view of the OBO Foundry is that the quality of an ontology is also - indeed primarily - determined by the accuracy with which it represents the pre-existing structure of reality [13]. Ontologies, from this perspective, are representational artifacts, comprising a taxonomy as their central backbone, whose representational units are intended to designate *universals *(such as *human being *and *patient role*) or *classes defined in terms of universals *(such as *patient*, a class encompassing *human beings *in which there inheres a *patient role*) and certain relations between them [14]. Here the terms 'universal' and 'class' are thus used to refer to entities in reality of the sorts denoted by the general terms in scientific and clinical textbooks. A class is, in mathematical terms, a set of individuals of one or other sort. Depending on whether the representational units designate correctly, the following distinctions are made:

• *Referring representational unit (RRU)*: an RU which is both intended to denote something and does indeed succeed in doing so.

• *Non-referring representational unit (NRU)*: an RU which, for whatever reason, fails to denote something.

• *Unrecognized non-referring representational unit (UNRU)*: an NRU which, although non-referring, is intended and believed to have a referent.

• *Recognized non-referring representational unit (RNRU)*: an NRU which was once intended and believed to have a referent, but which, as a result of advances in knowledge, is no longer believed to do so.

• *Representational unit component (RUC)*: a component of a representational artifact that is not intended by the artifact's authors to refer in isolation.

For an ontology of mental disease to be acceptable as an OBO Foundry ontology, it must adhere to the Foundry's principles and build further on relevant feeder ontologies, which in our present case are the Basic Formal Ontology (including the underlying Granular Partition Theory) and the Ontology of General Medical Science. For an ontology to be adequate as a foundation for the formal representation and classification of different types of mental disorders, its representational units need to be able to represent what it is for something to be a mental disorder. We use Pies' model of mental disorder as a test for the satisfaction of this latter criterion.

### Basic Formal Ontology

Basic Formal Ontology (BFO) is an upper-level ontology framework encapsulating best practices in the development of ontologies to serve scientific research. BFO is being used as basis for the creation of high-quality shared ontologies especially in the biomedical research domains. BFO is a realist ontology [15,16]. This means, most importantly, that representations faithful to BFO can acknowledge only those entities which exist in (for example, biological) reality; thus they must reject all those types of putative negative entities - lacks, absences, non-existents, possibilia, and the like - which are sometimes postulated as artifacts of specific terminologies or of associated logical or computational frameworks [12]. As an example, consider the following DSM-IV criteria for autistic disorder:

• failure to develop peer relationships appropriate to developmental level,

• a lack of spontaneous seeking to share enjoyment, interests, or achievements with other people,

• lack of varied, spontaneous make-believe play or social imitative play appropriate to developmental level.

Certainly there exist *reports *containing terms such as 'lack' or 'absence'; these reports are information artifacts, and as such they pose no problems for BFO. Processes such as *developing peer relationships *or *seeking to share enjoyment *or *spontaneous make-believe play*, too, can be represented in ontologies compatible with BFO - not, however, the *lack *or *absence *of such processes. Rather, the latter - often referred to as *negative findings *- have to be described exclusively by appeal to entities that do exist in reality, along the lines described in [17,18]. Lack of spontaneous make-believe play in some child (Jim) would thus be described in roughly this way: Not (Jim ***participant_of ***some instance of **make-believe play**).

BFO captures a small number of basic categories into which reality - or major portions thereof - is divided. First, it distinguishes *particulars *from *universals*; particulars are entities such as John, or the specific depression from which John (and he alone) has been suffering since last year; universals (represented by terms which are picked out in **Bold Small Capitals**), are repeatable entities, such as **Human Being**and **Depression**, which have particulars as their instances [12].

Second, BFO distinguishes within the realm of particulars between *continuants *and *occurrents*. Continuants are entities - such as John, his brain, his brain cells - that endure continuously through time while undergoing changes of various sorts. The domain of occurrents is made up of processes, entities which unfold over a certain time span through successive temporal parts or phases, including entities such as actions, and of the instantaneous boundaries of processes (beginnings and endings). Where continuants are extended in space and endure through time, processes are extended in both time and (in most cases) also space. What this means is that processes but not continuants can be partitioned along the time access: the occurrent entity that is your life, for example, can be divided into parts called 'your gastrulation', 'your childhood', 'your adulthood' and so on.

Third, there is the distinction between *dependent *and *independent *entities, the former being such that they cannot exist without some instance of the latter: John's height or weight, for example, cannot exist without John himself, and neither can his depression, or his feelings of remorse, or his bouts of anger. All processes are dependent entities: each process depends on at least one continuant.

BFO also distinguishes three major families of *relations *(depicted in ***bold italic face) ***between entities in the categories just distinguished: (1) <p, p>-relations: from particular to particular (for example: ***part of ***between John's brain and John; ***member of ***between Dr McX and the clinical staff of hospital Y; ***participant_of ***between Jim and his life); (2) <p, u>-relations: from particular to universal (for example: ***instance_of ***between John and the universal **Human Being**); and (3) <u, u>-relations: from universal to universal (for example: ***subkind_of ***between **Mouse**and **Organism**) [19].

### Granular Partition Theory

Granular partition theory is a framework for understanding the ways in which, when cataloguing, classifying, mapping or indeed diagnosing a certain portion of reality (*POR*), we divide up or partition this reality at one or more levels of granularity [20]. The resultant partitions are composed of *partition units *(analogous to the cells in a grid, to which labels may or may not be assigned), and the theory provides a formal account of the different ways in which such units can correspond or fail to correspond to the entities in reality towards which they are directed. It takes account also of the degree to which a partition represents the part-whole structure of the domain onto which it is projected, and of the degree of completeness with which a partition represents this domain.

Drawing on this framework, we have proposed in [21-23] a calculus for use in quality assurance of the complex representations created for clinical or research purposes, for example in coding of clinical trial data. The calculus is based on a distinction between three levels: [14]

(1) Level L1: the level of *reality *(for example, in the medical domain, the reality of pains, wounds, bacteria, on the side of the patient);

(2) Level L2: the level of *cognitive representations *of this reality, for example as embodied in observations and interpretations, as well as in beliefs, desires and other mental acts and states on the part of patients, clinicians, and others;

(3) Level L3: the level of *publicly accessible concretizations *of L2 cognitive representations in information artifacts of various sorts, of which ontologies, terminologies and Electronic Health Records are examples, as also are categorical systems such as the DSM.

The relation ***is_about ***can then hold between entities on levels L2 and L3 and corresponding level 1 entities.

By distinguishing these three levels we are able, for instance, to differentiate clearly between disorders and diseases on the one hand and diagnoses on the other. Disorders and diseases are L1 entities - they exist in first-order reality, on the side of the patient. Diagnoses, in contrast, belong to either L2 or L3, depending on whether they are formulated in a clinician's mind, or in an entry in an Electronic Health Record. Diagnoses are *about *particular disorder and disease instances. Papers published in scientific journals are (on our view) *about *corresponding universals or classes.

Representations in general are composed in modular fashion of sub-representations built out of *representational units *(representations none of whose proper parts are themselves representations), the latter being assumed by their authors to correspond to some *POR*. We note that:

1. each such representational unit is *assumed *by its author to be veridical, i.e. to conform to some relevant *POR *as conceived on the best current (scientific, or diagnostic) understanding (which may, of course, be based on errors);

2. several units may correspond to the same *POR*, for instance because they rely on different though still veridical *views or perspectives*, for instance at different levels of granularity (one thing may be described both as being brown and as reflecting light of a certain wavelength; one event may be described both as an event of buying and as an event of selling);

3. what units to include in a representation depends on the purposes which the representation is designed to serve.

The primary representational units in ontologies are terms from some natural or formal language that are assumed to denote universals [12].. Some terms, such as '**Bodily Feature**' in Table 1 (taken over from OGMS), do not denote directly, but are rather abbreviations standing in for disjunctions of terms that do denote in this sense.

**Table 1:** Disease-related definitions extracted and/or modified from the BFO-based Ontology of General Medical Science (OGMS) 25 and other sources

Generic Term	Level of reality	Type	Definition
**Bodily Component**	L1	*IC*	**Material Object* within ***or ***on the surface of ***an **Organism**, including **Anatomical Structures**, body flora, pathogens, toxins, and their combinations, potentially including also the organism as a whole [25,32]

**Bodily Feature**	L1	*POR*	**Bodily Component**, **Bodily Quality**, or **Bodily Process **[25]

**Bodily Process**	L1	*O*	**Process** in which at least one **Bodily Component **of an **Organism *participates ***[25]

**Bodily Quality**	L1	*DC*	**Quality* inhering in ***a **Bodily Component **[25]

**Clinical Phenotype**	L1	*POR*	a clinically abnormal **Phenotype **[25]

**Clinical Picture**	L2/L3	*POR*	a **Representation **of a **clinical phenotype **that is inferred from the combination of laboratory, image and clinical findings about a given **Organism **[25].

**Diagnosis**	L2/L3	*DC*	**Representation **asserting the presence of an instance of **Disease **in a given **Organism *resulting from ***an interpretive process that has as input a **clinical picture** of that **Organism **[25]

**Disease**	L1	*DC*	a **Disposition** (i) to undergo **Pathological Processes** that (ii) exists in an **Organism** because of one or more **disorders** in that **Organism **[25]

**Disease Course**	L1	*O*	the totality of all **Processes** through which a given **Disease** instance is ***realized ***[25]

**Disease Phenotype**	L1	*POR*	a **Clinical Phenotype** that ***is characteristic of ***a single **Disease **[25]

**Disorder**	L1	*IC*	A combination of **Bodily Components* of ***or ***in ***an **Organism** that is clinically abnormal [25,41]

**Observation**	L2	*DC*	**Cognitive Representation* about ***a **Bodily Feature* resulting from ***an act of perception [42]

**Pathological Process**	L1	*O*	a **Bodily Process** that is ***a manifestation of ***a **Disorder **[25]

**Phenotype**	L1	*POR*	a combination of one or more **Bodily Features* of ***an **Organism** determined by the interaction of its genetic make-up and environment [25]

L1 - L2 - L3: level of reality at which a portion of reality exists; *IC*: independent continuant; *DC*: dependent continuant; *O*: occurrent; *POR*: used either where a term is an abbreviation for a disjunction or where the term refers to an entity which instantiates some combination of universals.

Note that where terms in *ontologies *refer to universals, information artifacts of other sorts, including clinical records, clinical histories, reports of lab results, consist overwhelmingly of representational units that refer to instances, for example to patient-specific entities such as the specific feelings of depression experienced by patient McY during a certain period, or to McY's subsequent suicide attempts.

### The Ontology of General Medical Science

The Ontology of General Medical Science (OGMS) is an ongoing endeavor to facilitate inference across the boundaries of domain ontologies created to support research on Electronic Health Record (EHR) technology and on the integration of clinical and research data. It has been described by Jobst Landgrebe, former Co-Chair of the HL7 Vocabulary Group, as 'the best ontology effort in the whole biomedical domain by far' (personal email communication, Mon, Mar 22, 2010 at 11:45 AM). OGMS comprises representations of highly general universals in the domains of anatomy, physiology and pathology, of diagnosis and treatment, and of information artifacts such as clinical histories and lab test results [24,25]. It follows the principles of the OBO (Open Biomedical Ontologies) Foundry, of which the most important for our purposes are:

1. *avoid redundancy*: terms already included in existing ontologies should be imported where necessary in order to ensure that for each domain there should be convergence upon exactly one Foundry ontology; [26]

2. *exploit compositionality*: terms and definitions should be built up compositionally out of component representations taken either from OGMS itself or from external ontologies; [11]

3. *common architecture: *ontologies should use upper-level categories drawn from BFO together with relations unambiguously defined according to the pattern set forth in the OBO Relation Ontology; [19]

**Figure 1:**
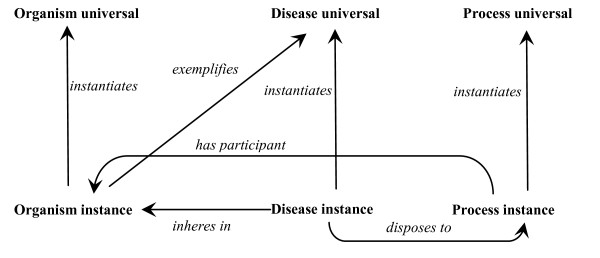
Ontological relations involving disease and bearer of disease

Figure 1 depicts in simple form the entities which exist on the side of a specific person when that person (an ***instance of *Organism**) has a disease. These include a **Disease** instance (this specific case of disease in this specific organism), which ***inheres_in ***that person, and the person thereby ***exemplifies ***the corresponding **Disease** universal. This **Disease** universal can be ***exemplified ***also by other persons. The **Disease **instance is a **Disposition** to processes of certain **process** universals, for example as manifested in the form of observable signs and symptoms.

Table 1 lists the terms from OGMS and related sources which we used to create definitions for entities in the realm of mental diseases. OGMS rests on the presupposition that the signs and symptoms associated with instances of disease, even those symptoms standardly qualified as being 'mental' - such as erroneous thoughts, difficulties in concentration, or auditory hallucinations - have in every case a physical basis.

Following OGMS we will use the term 'disorder' to refer exclusively to this physical basis on the side of the patient. To assert that something is *clinically abnormal *is then to state:

(1) that it is not part of the life plan for an organism of the relevant type (thus aging or pregnancy are not clinically abnormal),

(2) that it is causally linked to an elevated risk of pain or suffering or other feelings of illness or of death or dysfunction on the part of the organism, and

(3) that it is such that this elevated risk exceeds a certain threshold level.

Where, under the OGMS view, disorders are independent continuants (in simple terms, they are disordered parts of the patient's body), instances of the universal **Disease **are dependent continuants. Disorders are independent continuants because they are all material objects or aggregates thereof. Diseases are dependent entities because no disease can exist without some organism (such as a human being) to serve as its bearer. Diseases are continuants in that they endure through time while being associated with different sorts of changes, for example leading to more intense symptoms, or giving rise to new pathological structures within the body (e.g. progressively larger tumors). A disease is, more specifically, a continuant of the type *disposition*, defined in Basic Formal Ontology as: (1) a dependent entity that inheres in an independent continuant (its bearer) and is (2) that in virtue of which this bearer will participate in specific sorts of processes when certain (trigger) conditions are satisfied. Where is a disposition, there is thus a potentiality or tendency for certain things to happen. Examples are: the disposition of a human being to become sad when a loved one dies, or to become nervous when brought into a stressful situation. Human beings exhibit a wide variety of dispositions, some associated with health, others with disease. Diseases are special sorts of dispositions: they are dispositions realized in pathological processes [25].

In their capacity as dispositions, diseases are analogous to functions (e.g. of a bodily organ). Dispositions in general and functions in particular are *realized *in processes (as the function of your heart is realized *inter alia *in those processes which are pumpings of blood). Diseases may thus also be characterized as *dys*functions. Just as a particular function (for example of your heart) comes into existence at a certain time (the time when your heart first begins to exist), so a particular disease comes into existence at a certain time and gives rise thereafter to a typically complex series of processes, sometimes including manifestations that can be identified as signs and symptoms. These processes form (i.e. are part of) a second entity (an occurrent), for which we use the term *disease course*, and which we can think of as the *life *of the disease.

In turn, some of these processes may give rise to further continuants, such as a cyst or a redness of the skin, which come into existence as a result of processes such as inflammations. The relevant disposition can be instigated by a bodily aggressor such as a virus that enters the body, by biomolecular reactions that go wrong, or by processes which occur as the body defends itself against environment assaults. It can also come into existence as a result of a structural deviation in a person's genetic code resulting in a congenital disease.

The Tourette disease, for example, is characterized by both genetic markers and the presence of chemical abnormalities at the cellular and metabolic level in the brains of those who have the disease. One who has the genetic markers has the anatomical malformation and thus the disposition to the Tourette disease. Analogously, the influenza disease is marked by the presence of influenza virus infecting cells and reproducing, and is accompanied by numerous symptoms including fever, sore joints, and so on.

### Pies' account of the evolution of a disease entity

Pies proposes in [27] a 5-stage account of how our scientific understanding of a mental disease condition might evolve over time. His goal is a framework that will allow us to determine

*whether a condition represents, in the first place, dis-ease and, secondarily, whether it constitutes a specific disease, on a par with, say, bipolar I disorder? For example, how do we decide whether to consider "pathological bigotry" and "internet addiction" as specific mental disorders? *[27]

Following the view presented by Kendell in [28], Pies requires that for a mental disorder instance to exist - and note that Pies does not provide definitions for what he means by the terms 'disease' and 'disorder' - it must be the case that '*prolonged and severe suffering and incapacity in the affective, cognitive, or interpersonal-behavioral realms*' form part of the disease course. The evolution of our understanding of a mental disorder universal he then sees as passing through five stages:

• Stage 1 involves an acknowledgement of the patient's everyday experience of substantial and prolonged suffering and incapacity that is '*specified in terms of social and vocational impairment, impaired vital functions, and distortions in the phenomenological realm *(*feeling "totally worthless," "like I'm nothing"*)'. This must be acknowledged as an intrinsic element of having the condition and not simply as a consequence of society's punitive responses to the person's behavior.

• Stage 2 is marked by the acceptance of a general syndromal description of the condition, supported by evidence that the constituent signs and symptoms reliably 'hang together' over long periods and in geographically distant populations.

• Stage 3 is marked by the fact that the syndrome has been characterized by authoritative sources in terms of usual course, outcome, comorbidity, familial pattern, and response to treatment; there may also be preliminary data on pathophysiology and biomarkers, and a more specific understanding of the afflicted person's phenomenology.

• Stage 4 is marked by known pathophysiology, cause, a specific set of biomarkers, and in some cases an inheritance pattern for the condition (or for multiple conditions that become identified as separate entities only after Stage 2, as occurred for example in the case of anemia or diabetes).

• Stage 5 is characterized by the availability of a precise chromosomal and biomolecular etiology, and by a specification of the phenomenology, for all disease subtypes.

The relevance of the above to our argument will be explained in the section that follows.

## Methodology

Our methodology, which we have dubbed 'ontological realism' [12], is based on our previous work in building and assessing the quality of biomedical terminologies, coding and classification systems [21,23,29,30]. It stands in sharp contrast both to the terminological approach to ontology, which tries to identify the meanings of terms [31], and to the concept-based approach, which focuses on mere logical consistency. Our methodology starts out in every case with the attempt to identify the sorts of entities that exist in the salient portion of reality according to the best current scientific understanding. In our case here, it is the portion of reality described by Pies' model [27] and by the relevant parts of the 'Research Agenda for the DSM-V' [10] that is of relevance.

Pies' model has the advantage for our purposes that it does not rest on any assumption of incompatibility between biological and phenomenological data relating to what exists on the side of the patient. Rather it sees the distinction between the two sorts of data as resulting from complementary modes of analysis and observation. Pies is of importance also because he recognizes that 'best current scientific understanding' will take different forms at different stages of its development. Note that whether Pies is right in his analysis is not important here; what matters is whether our framework is able to represent what is believed to exist as expressed in his model.

The research agenda for the DSM-V, which serves as our second source, does not provide definitions for the terms 'mental disease', 'mental illness' or 'mental disorder', but gives some informal statements as to what these terms might mean, statements which can be supplemented by some associated passages found in the diagnostic criteria for the various types of mental diseases provided in the current version of the DSM.

BFO provides for our analysis an initial set of top-level representational units which are independent of any specific domain. OGMS, using BFO as foundation, expands the range of representational units to embrace the terms of general medical science. The first step in our analysis was thus the identification of relevant terms in the list of stages in the *evolution of a disease entity *recommended by Pies. For each of these terms, we then assessed whether it could denote either (i) one or other of the entities or relations described in section 2 or (ii) some configuration of such entities and relations. The question that is addressed in this step is thus, not the terminological question: *what do the identified terms in Pies' model mean?*, but rather the ontological question: *to what entities in reality do these terms refer? *We then, in the second stage, assessed for each of the identified entities whether they belong to the portion of reality described by either BFO or OGMS at the more general level, or to the more specific portion of reality to be described in our proposed Ontology of Mental Disease (OMD). For each entity at the level of BFO or OGMS, a corresponding representational unit had to be found, otherwise these ontologies would be marked by an unjustified gap [22]. For each entity pertaining strictly to the realm of mental health, we introduced a corresponding representational unit in OMD and attempted to create an associated Aristotelian definition [19] using representational units already defined in OMD, BFO, OGMS or in any other suitable external ontology. Here again we needed to check for the unjustified absence of representational units at the level of BFO and OGMS. The adequacy of BFO and OGMS as foundations for a formal representation of the entities in reality that the statements under scrutiny in Pies' model and the DSM-V research agenda attempt to describe was then measured in terms of any unjustified absences found.

## Results

We present here a set of terms and definitions representing the core entities involved in the phenomenon of mental disease, building further on BFO and OGMS (see Table 1). For each term, we indicate whether it refers

1. to an independent or dependent continuant universal (*IC *or *DC*),

2. to an occurrent universal *(O*)

3. to some portion of reality that is described through some logical combination of terms representing such universals (marked here with '*POR*').

**Figure 2:**
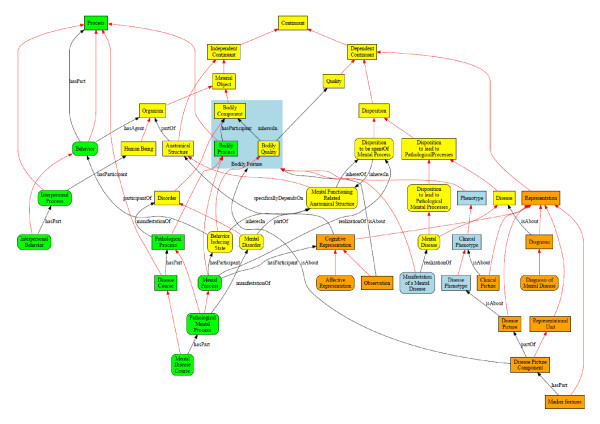
**Foundational entities for an ontology of mental disease**. Shape coding: terms in squared boxes are from feeder ontologies such as BFO and OGMS; terms in rounded boxes are specific to the Ontology of Mental Disease. Color coding: L1 continuants (first order entities) are depicted in yellow, L2/L3 continuants (representations) in orange; processes are depicted in green, abbreviations for disjunctions are in light blue. Red, unlabeled arrows indicate subsumption; black arrows are labeled according to the relation they depict, the label being either under or right from the arrows. Some obvious relationships have been left out to keep the figure intelligible.

Figure 2 contains an overview of the entities which we believe will need to play a foundational role in a future OMD alongside the entities already represented in BFO and OGMS.

To say that the patient has a mental disease is to say that there is some **Mental Disorder **instance in the patient. This is, in OGMS terms, the physical basis of the corresponding **Disease **instance. As a part of the organism it is, like the organism itself, an independent continuant (*IC*). This **Disease **instance is a certain *disposition *(*DC*), which inheres in the corresponding disorder in the patient, and is itself the *potentiality for *(and is *realized in*) instances of **Pathological Mental Process **(*O*) of certain process types, for example as manifested in the form of signs and symptoms. Typically, multiple patterns of such processes - multiple presentations, in other words, or what we shall officially call multiple **Disease Courses** (*O*) - will be associated with the same **Disorder **type, for example in reflection of the presence or absence of pharmaceutical or other interventions, of differences in environmental influence, and so forth.

In addition to the disorder-related entities are the various **Representations **of these and other entities, not only on the side of the clinician, but also - and this is a phenomenon of exceptional importance in the case of mental disease - on the side of the patient [14].

We assume, with OGMS, that **Cognitive Representations** - and other L2 entities such as beliefs, emotions and desires - have a physical basis (in the brain), in the relevant components of which there occur processes of certain sorts such as: activations of neurons, formation of synapses between cells, and flow of electrons (measurable, perhaps, by means of encephalograms). For convenience in what follows we shall call the corresponding physical components in the patient organism - components which are involved in both mental disease and normal cognitive functioning - '*mental functioning related anatomical structures*'.

A cognitive representation can participate in processes of different sorts. It can be *generated*: a cat crosses your path and causes your visual system to induce a configuration - a mental image - in your brain that allows you to describe the cat . It can be *sustained*: your mental image of the cat is kept in memory and allows you to describe the cat at later times. Or it is *modified*: you become aware that what you thought was a cat was not in fact a cat at all, but rather a raccoon.

A cognitive representation can in this way be ***about ***some other entity (the cat, again; or cats in general); or it can be, for example, a matter of hallucination, so that there is no entity which serves as that towards which it is directed (though of course the agent of the hallucinating act may believe that there is such an entity). On the realist strategy hallucinations and similar phenomena are then dealt with not as entities that are directed towards special kinds of objects (since there are, *ex hypothesi*, no such objects), but rather as special kinds of cognitive representations. Representations of this sort are, following the definitions provided earlier, non-referring representational units or what we shall henceforth refer to as *NRU*s.

### Mental health related entities that can exist in the absence of any mental disorder

• **Mental Functioning Related Anatomical Structure** (L1, *IC*) **=def. Anatomical Structure **in which there ***inheres ***the **Disposition** to be the ***agent of ***a **Mental Process.**

• **Behavior Inducing State** (L1, *DC*) **=def. Bodily Quality* inhering in ***a **Mental Functioning Related Anatomical Structure** which leads to **Behavior **of some specific sort.

• **Mental Process** (L1*, O) ***= def. Bodily Process** which brings into being, sustains or modifies a **Cognitive Representation **or a **Behavior Inducing State**.

• **Behavior **(L1, *O*) **=def**. a **Process **having **Processes **as parts in which an **Organism* participates as agent ***in response to external or internal stimuli and following some pattern which is dependent upon some combination of that **Organism**'s internal state and external conditions. (Derived from the Gene Ontology; see also [30]).

• **Interpersonal Process**(L1, *O*) **=def. Process **in which at least two **Human Beings **are agents.

• **Interpersonal Behavior** (L1, *O*) **= def. Behavior **having **Interpersonal processes **as parts, each involving the same instances of **Human Being.**

• **Representation** (L1,*DC*) = def. **Dependent Continuant **which ***is about ***a portion of reality.

• **Affective Representation **(L2, *DC*) **=def. Cognitive Representation** sustained by an **Organism **about its own emotions.

• **Cognitive Representation** (L2, *DC*) **= def. Representation** which ***specifically depends on ***an **Anatomical Structure **in the cognitive system of an **Organism**.

### Mental disorder related core entities

• **Mental Disorder **(L1, *IC*) **=def. Disorder *in ***a **Mental Functioning Related Anatomical Structure.**

• **Pathological Mental Process** (L2, *O*) **= def. Pathological Process** which is the ***manifestation of ***a **Mental Disorder.**

• **Mental Disease **(L1, *DC*) **= def**. a **Disease** which is a **Disposition** to undergo **Pathological Mental Processes.**

• **Mental Disease Course** (L1, *O*) **= def. Disease Course** of a **Mental Disease.**

• **Manifestation of a Mental Disease **(L1, *POR*) **= def**. a **Bodily Feature** of an **Organism **that is (a) a deviation from clinical normality that is the ***realization of ***a **Mental Disease **and is (b) observable.

### Diagnosis related core entities

• **Diagnosis of Mental Disease **(L2/L3, *POR*) **= def. Diagnosis **asserting the presence of an instance of **Mental Disease** in a given **Organism.**

• **Disease Picture **(L3, *DC*) **= def. Representation** of a **Disease Phenotype **concretized in some information artifact.

• **Disease Picture Component** (L3, *POR*) **= def. Representational Unit** which is a part of some **Disease Picture** and which either (1) represents (or is believed to represent) a **Bodily Feature **that is believed to be manifested in the **Disease Phenotype **represented by the **Disease Picture** of which it is a component or (2) expresses a negative finding (as discussed in [17]).

• **Collection of Marker Features for Disease X **(L3, *DC*) **=def. Representation **which is a collection of **Disease Picture Components **which are characteristic for **Disease X **(where '**X**' serves as placeholder for some disease name).

## Discussion

### The purpose of definitions

The definitions formulated in the foregoing are not definitions for familiar words or terms in their familiar usage. Thus in particular our proposed definitions for the terms 'mental disease', 'disease course', and 'disorder' clearly do *not *capture the uses of these terms that are predominant in the literature. This is for multiple reasons, not the least of which is our striving for clarity and consistency - where clear and consistent distinctions between these and cognate terms are not in general drawn. That this is a problem is recognized already in the DSM, and in attempting to solve it we are in fact addressing a challenge formulated by the DSM itself. What is essential from our point of view - and this goes to the very heart of ontological realism [12] - is first, to point out the salient types of entities that exist, and then to associate with each of them a name that, in conjunction with its definition, allows the reader to understand what *we *mean by this term both here and in future work. We do not suggest that all the terms proposed in the above should be used by clinicians, although moves in this direction would help to make medical jargon less ambiguous (while at the same time potentially bringing other costs). What is more important is a broad recognition of the existence of the types of entities denoted by these terms, since without this broad recognition we will not achieve the sort of terminological clarity that is needed for computational purposes such as integration of mental health data with biological and other sorts of data. Finding better terms for the entities in question is, in this light, a secondary issue.

In this connection it is important to bear in mind that definitions for terms, and for representational units in general, may serve different purposes. These purposes are: [32]

P1: to specify the conditions that must be satisfied for a term to be an acceptable designator for a given entity in some given community. Such definitions often reflect decisions concerning more or less arbitrarily selected thresholds in relation to measured quantities or qualities marked by gradiants. Examples are the definition for 'severe mental retardation' in ICD-10 in terms of *an IQ of less than 34*, or of 'chronic pain', defined as *pain lasting longer than 3 months*;

P2: to specify what is characteristic of particulars that instantiate a certain universal, for example: disorder = def. a combination of bodily components of or in an organism that is clinically abnormal;

P3: to demarcate groups by specifying characteristics that their members must exhibit, for example: depressions in patients that participated in clinical trial X.

P1 definitions are essentially terminological. Often they are associated with decisions of a more or less arbitrary sort. The definition for 'chronic pain' excludes the use of this term for pains lasting less than 3 months. This does not mean, however, that a pain in a specific patient that has already lasted for 90 days *becomes *a chronic pain exactly one day later. A chronic pain was, ontologically, chronic already from the very beginning, even though this fact was unknown to any observer. Similarly, there are terminological, but not medical, arguments for claiming that two patients with otherwise similar clinical pictures suffer from instances of distinct disease types because one patient has a disease picture component 'IQ = 33' while the other has component 'IQ = 35'.

P2 and P3 definitions can help in determining whether a given particular is to be classified in a given way. P2 definitions fulfill this role at the level of universals (corresponding to what is the case in reality 'following the laws of nature'), while P3 definitions reflect demarcations made by decree.

### Application of definitions

The definitions pertaining to diagnostic criteria that are currently in use, for example in the DSM, are of types P1 or P3. The goal is, of course, for all definitions to be of type P2. Such definitions should specify the necessary and sufficient conditions for some entity to be an instance of the universal that is being defined.

The definitions that we provide in our framework require some familiarity with the practical dimensions of the realism-based approach to ontology if their implications are to be properly understood. As an example, one might wrongly assume that the brain itself is a *mental functioning related anatomical structure *as defined here, and that therefore any brain disorder is also a mental disorder. This, however, is not the case, since there are brain disorders such as aneurysms which are not mental disorders. Many types of mental disease are in addition such that the underlying etiology and pathophysiological mechanisms are not yet fully understood, although science is, we believe, making impressive advances in this regard (albeit with occasional reverses). Almost all theories of mental disease do indeed resort in some way to abnormalities in *parts *of the brain. But the careful reader will have noticed that in our definitions it is nowhere specified in what parts of the body mental functioning related anatomical structures are assumed to exist. It is up to neuroscientists to specify which anatomical structures are *mental functioning related *in the sense here at issue and as such are the ***agents of ***instances of **Mental Process **in which a **Cognitive Representation **or **Behavior Inducing State **bring into being. If, in the future, it were discovered that, for example, cells in the sense organs play a role in the generation, maintenance or modification of cognitive representations, then no change to our framework would be required in order for this new information to be accommodated.

As a further example, it has been shown that patients with major depression exhibit an increased number of cells in the limbic thalamus [33]. Even if it were discovered that the cells in question were causally involved in the corresponding dispositions on the side of the patient, however, it would still be wrong to infer that the *entire *limbic thalamus is a mental functioning related anatomical structure under the definition provided. If this sort of inference were valid, then the entire human body would deserve the same status. It is for this reason, too, that we do not specify in our framework that if part of an anatomical structure is a mental functioning related anatomical structure, then the entire structure of which it is part is also a structure of this type.

### Mental diseases, disorders and illnesses

The universals **Cognitive Representation** (including **Affective Representation** as subtype) and **Behavior Inducing State** play a key role in our ontology. For it is by reference to these universals that we define the universal **Mental Process**, reference to which is used in turn to describe the universal **Mental Functioning Related Anatomical Structure**, instances of which are the seat of mental diseases.

Building on this basis, we can now describe a *person with canonically good mental health *as one who exhibits a close conformity of perception, emotion, and behavior both among themselves and in relation to the surrounding real-world environment. In other words, in a mentally healthy person, perceptions and emotions coincide with reality and behavior is appropriate to its context. Mental disease is then, drawing on the OGMS perspective on disease in general, a clinically significant deviation from mental health as so defined, a deviation that hampers the bearer in his or her mental well-being.

Our view of mental disease thus corresponds in part with Derek Bolton's account, which sees mental disease as involving a "*radical failure of intentionality*" [6], where 'intentionality' refers to the capacity of our brain to generate cognitive representations that are about or stand for things, properties and states of affairs in reality [34].

In a mentally healthy person, intentionality simply works. If an individual's being *healthy *depends in this way upon the degree of correlation of perceptions, emotions and behavior to reality, then a *mental disease *exists where there is impairment due to a disorder in the intentionality-related and thus mental functioning related parts of the patient's body.

### Mental diseases and diagnostic criteria

Since at least the emergence of DSM-III, mental disorder diagnosis and treatment has rested on the assumption of two underlying needs (1) to identify physiological/neurological etiology and (2) to employ evidence-based approaches to treatment. Because of the history of the DSM, however, the classification of disorders remains a hodgepodge, which means that science that would support the realization of the two identified needs is not able to benefit from data coded on the basis of existing revisions of the DSM.

The initial challenge for developing an ontology of various subtypes of mental diseases is that of sorting out an inventory for the higher-level universals **Mental Disease**, **Disorder** and **Disease course **along the lines set forth above. For in order to be able to classify the different types of mental disease, one must know what a mental disease *is*, and what distinguishes mental disease from, say, non-mental disease. While some have argued that there are no such universal higher-level types - for example because what counts as 'mental disease' is entirely contingent upon culture - research on the basis of the prevailing biomedical model of mental illness is providing increasing evidence to suggest otherwise. Certainly there are, for a number of different reasons, differences from culture to culture as concerns what is counted as *clinically significant *in the realm of mental disease. But just as influenza infection causes the same type of disease throughout the world even in spite of cultural differences in the way this infection and its consequences are described, so, we would argue, schizophrenia and other mental illnesses exist as commonly recognized types of dysfunction even in spite of cultural differences in the categorical schemes used to describe it. The OGMS-based definition of mental disease that we have proposed accounts for the existence of these universals, and we believe that it will ultimately help to replace faulty categorical schemes with a new, and more robust, account of disorder, disease and disease course of a sort that will enable a more robust bridge between clinical and biological data.

This optimism needs however to be tempered by the perspective that our understanding of the etiology of diseases in general and of mental diseases in particular is and will continue to be subject to rapid changes as a result of advances in the relevant biomedical sciences and associated assay technologies. Thus we need always to bear in mind that what is originally perceived as being a single disease type might later turn out to be a plurality of distinct diseases whose instances manifest in similar fashion despite distinct underlying etiologies. We are confident, however, that our framework provides resources for such changes to be accurately represented, and this precisely because of (1) the distinctions we have drawn between (a) disorder, disease, disease course and disease phenotype on the side of the patient, and (b) the *representations thereof *for example on the side of the clinician, and (2) our view that diseases are *dispositions*, and therefore such that the nature of their manifestations is dependent on the circumstances which bring them to realization. It is precisely the lack of such ontological distinctions in traditional descriptions of 'disease' that leads to the errors, for example of a type which involve focusing not on 'disease' but rather on 'diagnosis' - for example because a patient presents with symptoms whose pathophysiological basis is not yet fully understood.

The framework presented here can help to avoid such errors because its basis in OGMS allows it to do justice in consistent fashion to a range of distinctions not easily captured in traditional approaches, including:

• the distinction between **Mental Disease **instances that do and those do not lead to instances of** Mental Pathological Processes**,

• the fact that a given **Clinical Picture **instance may reveal only certain parts of the corresponding **Mental Disease Course**, or it may reveal only certain untypical aspects of the canonical **Disease Course** for a disease of the given type **-**for example because it was created before certain diagnostic tests or procedures became available,

• the fact that phenotypically similar **Mental Disease Course **instances may be the result of dissimilar **Mental Disease **instances.

And because these distinctions can be made, it is possible for the framework to create multiple different classifications which yet remain mutually comparable, potentially including multiple classifications which can be shown to be equally valid from an ontological perspective. This mutual comparability is important above all because it allows data collected on the basis of the different classifications to be exploited for research purposes - for example in the evidence-based revision of the DSM.

### Reformulating Pies' model

To see how this works, we show how the framework allows in particular the reformulation of Pies' model in such a way that all five stages are formulated in a consistent fashion. The central organizing feature of his account is then seen to lie in the fact that at each stage there are **Observations** of **Bodily Features** at different levels of granularity that are relevant to the **diagnosis**of **mental disorder**. At least one of these phenomena must itself either be, or be a cause of, something that can be viewed from the perspective of the patient himself as a matter of '*prolonged and severe suffering and incapacity in the affective, cognitive, or interpersonal-behavioral realms*'. Pies provides accounts of patients who describe themselves as feeling, for example '*totally worthless*', '... *like I'm nothing*'. It is important to be clear about the complex nature of the goal Pies has set himself in this connection, a goal which obliges him to skirt very close to the edge of definitional circularity. For he is seeking to establish when, in the course of the development of our scientific knowledge, it is justified to assert that a mental disease (by which we understand him to mean a mental disease *type *in the categorical sense) exists and is exemplified by multiple patients. The complexity arises because part of such justification will involve appeals to experiences on the parts of patients or potential patients which themselves refer to, or are directed towards, phenomena which might be classified as mental disease.

Pies' view is that the discovery of a mental disease type starts with the acknowledgement *by the patient *of his or her suffering in the affective, cognitive, or interpersonal-behavioral realm and that such suffering is '*an intrinsic element of the condition*'.

A first stage diagnosis does not as yet involve any assertion as to what specific type of mental disease a patient is suffering from. Rather it conveys a belief (L2) or statement (L3) on the part of the clinician to the effect that the patient has some instance of some mental disease.

Pies' second stage involves the recognition that a multiplicity of patients exhibit a similar **Clinical Picture** as defined in Table 1. In the ideal case, these **Clinical Pictures **(L2/L3) are accurate representations of underlying **Clinical Phenotypes **(L1); but things may go wrong, specifically when different observers are independently describing distinct patients in the absence of standardized diagnostic tools and of associated classificatory resources. Patients with a similar **Clinical Phenotype** may then become associated with **Clinical Pictures** of distinct sorts, or patients with distinct **Clinical Phenotypes **may be believed to exhibit a similar **Clinical Picture**. Furthermore, at this stage, according to the Pies model, we have only a general syndromal description of a condition. Thus it is not yet possible to infer that, because patients exhibit a similar **Clinical Phenotype** they also exhibit any common **Disease Phenotype** (i.e. a phenotype characteristic of some single disease).

Note that, for something to be a **Disease Picture**, it is not required that all the **Disease Picture Components** included within it are correctly so included. Each **Disease Picture Component** belongs to one of the categories identified for representational units, although it might not be known at any given time which one precisely. We can however assume that, when a responsible authority publishes a **Disease Picture**, then it is assumed by that authority that all **Disease Picture Components** are Referring Representational Units (*RRU*s).

For stage 3, Pies' 'course' corresponds to OGMS' **Disease Course**, and his 'usual course' translates into OGMS' **Disease Phenotype** (Table 1). Entities that correspond to Pies' notions of 'outcome', 'comorbidity', 'pathophysiology' and 'response to treatment' are all examples of the processes that form part of the **Disease Course **for any given mental disease. Of course, for any subtype of **Mental Disease**, there are corresponding subtypes of such processes ranging from the appearance of hallucinations, mood changes, irrational thinking on the one hand, to molecular and cellular processes of various sorts on the other.

Stage 3 refers also to the presence of a familial pattern, something that can become apparent from data collections about sibling relations between patients who exhibit a **Mental Disease Course** of the same type. What Pies refers to as 'biomarkers', and 'a more specific understanding of the afflicted person's phenomenology' are represented by means of our **Disease Picture Component** and **Collection of Marker Features**. Pies' later stages thus do not require the introduction of further types of entities at higher levels, but rather the accumulation of more instance data, and a deepening of the taxonomies of processes and bodily components.

## Future work: towards a full-fledged ontology of mental disease

In this paper, we focused on the question: *what is a mental disease*? Our answer to this question will, in the next stage, be expanded into an ontology of the various mental diseases which form the subtypes of the universal **Mental Disease**. This will involve a taxonomy not only of such subtypes but also of associated physiological and pathological processes (thereby in part linking to other ontologies such as the Gene Ontology [21]). It will require also evidence-based assertions about which of these processes are parts of the disease courses corresponding to the mental diseases of the different distinguished subtypes. Our long-term objective is to develop an ontology of mental disease along these lines. This ontology should in addition accomplish the goals of (1) serving as a bridge ontology with other classifications of mental disorders like the ICD, (2) enabling field and clinical data collection through electronic medical records that would be interoperable with other existing modes of diagnoses and treatment, and (3) solving the following problems with DSM as summarized in the DSM-V Research agenda [35]:

a) severely ill inpatients often meet criteria for more than one DSM-IV personality disorder, so that according to this system there would be a high rate of co-morbidity even where there is no medical or etiologic reason for assuming the co-existence of two or more separate disorders in the mentioned patients;

b) many outpatients do not meet the criteria for any of the specific categories identified in DSM-IV;

c) the diagnostic thresholds separating what is normal from what is disordered are subject to frequent revision, so that it is sometimes as if given disorders would appear and disappear in course of time, and this both at the level of the diagnoses formulated for individual patients and at the level of disease types.

These problems have been used as arguments to favor an alternative, so-called *dimensional *approach, according to which disorders would be described in terms of points or regions along a number of 'diagnostic continua'. Where, for instance in the case of depression, the categorical school postulates that patients who are both depressed and anxious as instantiating two disorder types, the dimensional school regards them as cases of 'anxious depression' [36], conceiving depression and anxiety as overlapping regions within a multidimensional continuum whose dimensions are defined in terms of different sorts of observable variations, examples being severity or frequency of occurrence. Thus whereas the categorical approach requires assessments to be made whether a patient exhibits a symptom or configuration of symptoms which is considered to be pathognomonic for some mental disorder, the dimensional approach highlights the necessity of assessments in terms of, for instance, the severity of a symptom or the frequency with which it is manifested. Reconciling the dimensional and categorical views, another request made in the DSM-V Research Agenda, is thus another goal that we hope to reach with the framework here proposed.

One issue is that for a number of the diagnostic categories mentioned in DSM-IV we still lack any scientific base for the understanding of the corresponding disorder types. As an example, it is part of the DSM Research Agenda that a definition of 'mental disorder' should be such (a) that it '*can be used as a criterion for assessing potential candidates for inclusion in the classification, and deletions from it*' and (b) that there should be '*at least no ambiguity about the reason that individual candidate diagnoses are included or excluded*' [10]. It is stated also that if a satisfactory definition of 'disease' can be formulated at all, then it should take into account both biomedical and sociopolitical considerations.

We have argued that, to achieve this needed scientific basis, our realist approach must be supplemented by what we have identified as the referent tracking strategy [37] in the treatment of patient information. This strategy provides a formal mechanism for linking ontologies to the EHR and other patient data repositories, providing the latter with the needed anchor in the reality on the side of the patient. Unfortunately, the widespread use of entity-relationship and object-oriented modeling approaches still presents an obstacle to effective progress on this front, since these approaches are designed to support the construction not of representations of reality but rather of what have been called 'models of information'. And while this contrast is not yet widely understood [38,39], we are beginning to see new applications of the referent tracking approach outside the realm of its original designers [40].

## Conclusions

By using the realism based approach we have been able to identify certain confusions and conflations in the clinical terminology around mental disease, disorder, and illness. To bring clarity, we presented an ontology resting on the identification of mental diseases as *dispositions *which exist because of underlying *disorders *in mental functioning related anatomical structures and which become realized in *pathological processes *that make up the relevant *disease course*. By being rooted in Basic Formal Ontology and in the Ontology of General Medical Science our ontology is fully compatible with other ontologies that adhere to the principles of the Open Biomedical Ontology Foundry. A further strength is that our approach is not based on any specific theory of mind, nor does it commit us to either the categorical or dimensional view on mental disease. Although we believe that it is possible to express by means of our framework such theories of the mind as might be advanced, future work in this direction will be required in order to put this claim to the test. Such work will include a complete re-representation of the entities described by the diagnostic criteria of both ICD and DSM.

Devising an ontology of mental disease consistent with other biomedical ontologies poses obvious potential benefits. Among these are: better communication among disciplines, coordinated diagnosis and treatment, as well as clinical trials and drug development aided by interoperable biomedical and psychiatric ontologies. Of course, the ultimate potential benefit, if our proposals are soundly based, will be better mental health for patients.

## Competing interests

The authors declare that they have no competing interests.

## Authors' contributions

WC analyzed the source materials, performed an ontological analysis of the domain described therein, and proposed a first list of entities to be represented in an ontology for mental disease with corresponding terms and definitions. Both authors then refined the definitions until there was full agreement. Both authors read and approved the final manuscript.
